# Mobile ECMO for inter-hospital transport of pediatric patients: experience from 22 cases

**DOI:** 10.3389/fped.2025.1664454

**Published:** 2025-09-24

**Authors:** Yufan Yang, Xiangni Wang, Xiulan Lu, Xinping Zhang, Jiaotian Huang, Zhenghui Xiao

**Affiliations:** ^1^ Department of Intensive Care Unit, The School of Pediatrics, Hengyang Medical School, University of South China (Hunan Children’s Hospital), Changsha, Hunan, China; ^2^ Department of Intensive Care Unit, The Affiliated Children’s Hospital of Xiangya School of Medicine, Central South University (Hunan Children’s Hospital), Changsha, Hunan, China; ^3^ Department of Internal Medicine Teaching and Research Office, Hunan Traditional Chinese Medical College, Zhuzhou, Hunan, China

**Keywords:** ECMO, inter-hospital transport, mobile ECMO, children, critically ill patients

## Abstract

**Objective:**

To summarize the management experience of inter-hospital transport of critically ill children with extracorporeal membrane oxygenation (ECMO) in our hospital and provide evidence for the mobile ECMO for inter-hospital transport of pediatric patients.

**Methods:**

Critically ill patients treated with ECMO transported to our hospital from January 2020 to July 2025 were included in this study and analyzed general information, disease types, cannulation methods, ECMO transport distances, patient conditions before and after ECMO deployment, complications during the transport, and outcome. The lesson was drawn up regarding individual protection, transport procedures, transport equipment, teamwork, monitoring during transport, and quality control, providing an evidence-based foundation for the mobile ECMO for inter-hospital transport process of critically ill children.

**Results:**

A total of 22 critically ill pediatric patients were successfully transported to our hospital supported with ECMO by ambulance. The oldest child was 13-years-old, and the median age of the cohort was 76.00 (19.00, 132.00) months. The primary diseases included acute respiratory distress syndrome (ARDS), fulminant myocarditis, avian influenza, heart failure, and persistent pulmonary hypertension of the neonate. The median transport distance was 180.00 (134.00, 233.00) km, and the patients had no complications during the transport. Subsequently, 17 patients recovered and were discharged from the hospital. Five patients developed with multiple organ failure soon after the separation of ECMO. The ECMO duration was 126.50 (83.00, 155.00) h. No infection in any medical staff and nursing staff.

**Conclusion:**

The availability and safety of mobile ECMO for inter-hospital transport of critically ill children could be improved with the support of a well-equipped technical team in a time-effective manner, saving patient lives.

## 1 Introduction

Extracorporeal membrane oxygenation (ECMO) is a life-saving measure used to support heart and lung function and strive for time for other treatments (1). Some studies have shown (2) that ECMO can save lives of patients with pneumonia caused by novel coronavirus in combination with cardiopulmonary failure. Kendirli et al. (3) stated that mobile pediatric extracorporeal membrane oxygenation transport teams may provide safe aircraft and ground vehicle transportation in high-risk patients with acute cardiogenic shock bridging to survival or long-term circulatory support.

Due to the treatment's complexity, risk, cost, and complications, ECMO needs to be carried out in an experienced medical center. However, given the current economic situation in China, it is impossible to equip all hospitals with ECMO machines and master this technology, thus making it necessary to initiate ECMO at local hospital and then transport patients to tertiary hospital with the necessary resources for follow-up treatment.

Children who undergo mobile ECMO for inter-hospital transport are in critical condition and exposed to various conditions, such as distance, time, equipment, and transport teams during the transport process, which requires high demands on the safety and feasibility of mobile ECMO for inter-hospital transport. The mobile ECMO for inter-hospital transport in the West has been proven safe and feasible (4–8), but that in China is still in its infancy. The transport of pediatric patients treated with ECMO has become challenging in China. The ECMO transport team in our Department of Pediatric Intensive Care Unit (PICU) of Hunan Children's Hospital in Changsha City has successfully performed mobile ECMO for inter-hospital transport of 22 critically ill patients. Thus, the experience of managing inter-hospital ECMO transport for pediatric patients is valuable in China.

## 2 Methods

### 2.1 General information about the patients

Patients who received ECMO (MAQUET, Sweden, GETINGE) in our hospital from January 2020 to July 2025 included 10 boys and 12 girls, with a median age of 76.00 (19.00, 132.00) months. The oldest child was 13-years-old. The primary diseases included acute respiratory distress syndrome (ARDS), neonatal respiratory distress syndrome (NRDS), fulminant myocarditis, avian influenza, heart failure, and neonatal pulmonary hypertension. Two children suffered cardiac arrest before ECMO treatment and were given external chest compressions, and all 22 children were treated with mechanical ventilation. This manuscript has obtained human research ethics approval from Ethics Committee of Hunan Children's Hospital in accordance with the Declaration of Helsinki, approval number: HCHLL-2023-121.

### 2.2 Transport of patients supported with ECMO process

#### 2.2.1 Preparation of physicians

The ECMO-multidisciplinary team in our hospital consists of medical and nursing staff from nine departments, including PICU, Cardiothoracic Surgery, Anesthesiology, Cardiology, Radiology, Ultrasound, Pharmacy, and Laboratory. The ECMO team members for each transport included two pediatric doctors from PICU, one pediatric cardiothoracic surgeon, one pediatric anesthesiologist, one nurse from PICU, one nurse from the transport center, and two drivers in two ambulances.

#### 2.2.2 Preparation of patients for teleconsultation and instruments

When the patient's condition worsened, the physician from PICU of the local hospital assessed the condition, communicated with our experts, made a preliminary agreement with the patient's family on the intention of transport, and then informed the PICU of our hospital via telephone. After the chief physician with extensive care and ECMO management experience reassessed the patient's condition, the doctor at local hospital informed our ECMO team to bring complete set of ECMO equipment to prepare for departure to the local hospital. The relevant transportation department was contacted for assistance with route planning to save patients as soon as possible.

#### 2.2.3 ECMO implementation

Upon arrival at the local hospital, the ECMO team reassessed the patient's condition, including cardiopulmonary function and vascular status, and selected appropriate cannulation method. Also, staff and equipment were assigned, the patient's family members were communicated with, and relevant medical documents were signed before the ECMO operation. The local hospital staff was responsible for the resuscitation and medication administration of the patient with proper documentation. The ECMO team established the ECMO vascular access under strict sterility according to the patient's condition. The nursing staff installs and commissions the ECMO pipelines and assists the physician by delivering the consumables and monitoring the activated clotting time. The cannulation approach is surgical approach.

#### 2.2.4 Transport of patients supported with ECMO

During the transport to the ambulance, medical members carry the patient in a flat position by a trolley, ensure that all the tubes are unobstructed without pulling or bending, and pay close attention to the child's vital signs. During transport process, respiration, heart rate, blood pressure, body temperature, pupil size, oxygen saturation of the patient, peripheral circulation status, speed and flow matching of ECMO equipment, the color change of the blood in the circuit, the oozing of the cannulation site, and any abnormal changes in the machine are monitored. Furthermore (9, 10), the dose of vasoactive drugs should be gradually reduced according to heart rate and blood pressure in patients undergoing veno-arterial ECMO. The transport can be started 30–60 min after the patient's condition is stable, the ECMO flow is stable, and there is no evidence of internal bleeding, such as a significant drop in hemoglobin level (11). Another study showed that patients experienced decreased ECMO flow and blood oxygen saturation during transport, mainly due to hypovolemia, which improved after rehydration and position adjustment (12). In 57 children with ECMO reported by Fouilloux et al. (5), no adverse events occurred during ECMO transport. Herein, the 22 children transported to our hospital also had no adverse events during the transport. Criteria for V-A ECMO of ARDS pediatric patients weaning: the ECMO flow rate was gradually reduced to 10–20 ml/kg per hour, with the blood flow decreasing to 20% of the full flow rate. The arterial blood gas parameters and cardiac function were good. Criteria for V-A ECMO of fulminant myocarditis pediatric patients weaning: (1) Improvement in cardiac function in children with fulminant myocarditis [Cl ≥ 3.6 L/(min·m^2^), LVEF ≥ 40%–45%]; (2) No malignant arrhythmia on 24-hour dynamic electrocardiogram; (3) ECMO pump blood flow has decreased to 15%–20% of ideal cardiac output, and ECMO can be weaned if indicators such as normal blood pressure, blood lactate <2 mmol/L, and ScvO_2_ 65%–75% can be maintained for less than 6 h with the discontinuation or only use of small doses of vasoactive/positive inotropic drugs.

## 3 Results

**Figure 1:**
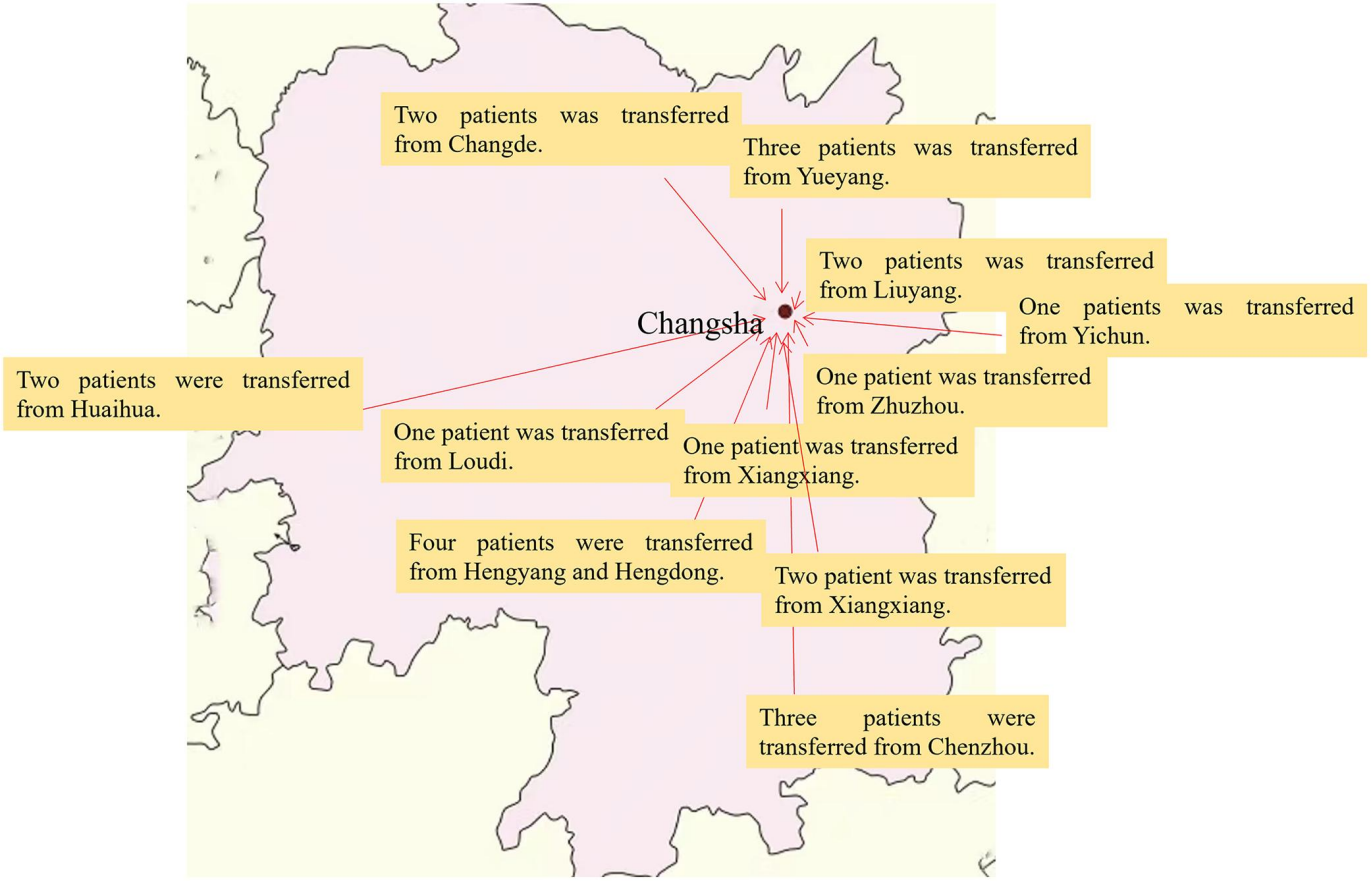
Inter-hospital ECMO transfer map of critically ill children in Hunan Province. This map is Hunan Province, Changsha is the capital city of Hunan Province.

All 22 patients arrived safely at our hospital. The transport distance was 77–354 km (Figure 1), with median transport distance of 180.00 (134.00, 233.00) km. Patients had stable respiratory and circulatory status without any complications during transport. Complications during transportation included catheter-related bleeding, neurological complications, gastrointestinal bleeding, thrombosis, hemolysis, liver dysfunction, DIC, and severe thrombocytopenia. The median ECMO duration was 126.50 (83.00, 155.00) h. Subsequently, 17 patients were discharged after improvement. Five patients developed multi-organ failure soon after separation from ECMO and died eventually. The general information of the 22 pediatric patients is summarized in Table 1. The survival rate of fulminant myocarditis is 82%, the survival rate of fulminant myocarditis is 75%, the survival rate of fulminant myocarditis is 66.7%.

**Table 1:** Summary of the general information of the children.

No.	Sex	Age (Months)	Original disease	ECMO type	Outcomes	Weight (Kilogram)	Cannula size (internal jugular vein, arteria carotis communis)	Complications
1	Female	82	Fulminant myocarditis	VA	Survival	27	19FV, 17FA	None
2	Female	77	Fulminant myocarditis	VA	Survival	25	19FV, 17FA	None
3	Female	24	ARDS	VA	Survival	11.5	17FV, 14FA	None
4	Male	19	ARDS	VA	Survival	9.5	17FV, 14FA	None
5	Male	4 days	NRDS	VA	Survival	3.1	10FV, 8FA	None
6	Male	111	Fulminant myocarditis	VA	Death	28	19FV, 17FA	None
7	Female	7 days	Meconium aspiration syndrome	VA	Survival	4.8	10FV, 8FA	None
8	Male	132	Heart Failure	VA	Survival	40	Femoral vein: 23FV Femoral artery: 21FA	None
9	Female	72	Avian influenza: H5N6	VV	Death	17	17FV, 14FA	None
10	Male	3	Severe pulmonary valve stenosis	VA	Survival	5.7	10FV, 8FA	None
11	Female	118	Fulminant myocarditis	VA	Survival	35	19FV, 17FA	None
12	Male	142	Fulminant myocarditis	VA	Survival	54	Femoral vein: 23FV Femoral artery: 21FA	None
13	Female	148	Fulminant myocarditis	VA	Survival	35	19FV, 17FA	None
14	Female	147	Fulminant myocarditis	VA	Survival	55	Femoral vein: 23FV Femoral artery: 21FA	None
15	Male	2 days	NRDS	VA	Survival	2.5	10FV, 8FA	None
16	Male	164	ARDS	VA	Death	58	Femoral vein: 23FV Femoral artery: 21FA	None
17	Male	19	ARDS	VA	Survival	12	17FV, 14FA	None
18	Male	20	Fulminant myocarditis	VA	Survival	14.5	17FV, 14FA	None
19	Female	132	Fulminant myocarditis	VA	Death	35	19FV, 17FA	None
20	Female	104	Fulminant myocarditis	VA	Survival	26	19FV, 17FA	None
21	Female	1 day	NRDS	VA	Death	3.2	10FV, 8FA	None
22	Female	75	Fulminant myocarditis	VA	Survival	21	19FV, 17FA	None

ARDS, acute respiratory distress syndrome; NRDS, neonatal respiratory distress syndrome; VA, veno-arterial; VV, veno-venous; ECMO, extracorporeal membrane oxygenation.

## 4 Discussion

This article is about how to overcome the difficulties, and how to use ECMO to transport critically ill children from local hospitals to tertiary hospital.

ECMO is an extracorporeal life support technology that can effectively replace the patient's respiratory and heart functions and maintain the oxygen supply to organs. Timely ECMO treatment for critically ill patients with reversible conditions is crucial to helping patients through the most critical moments. Moreover, Broman et al. (13) showed that 13 patients who died before ECMO installation could be ascribed to the issue of treatment delay and the importance of immediate alerts from local hospitals in case of hemodynamic or respiratory deterioration, highlighting the need for hospitals to contact ECMO centers at the earliest. However, due to the complexity of the technology and equipment, hospitals at the prefecture level in Hunan Province were not equipped with pediatric ECMO instruments, which made it difficult for us to transport the children, and increased the risk of coronavirus infection to the healthcare staff due to the varying levels of epidemic control. Therefore, strict isolation conditions are required for ECMO operations.

Some studies (13, 14) have concluded that a specialist anesthesiologist in the pediatric ECMO transport team is required. The process of cannulation in pediatric patients differs from that of adults. Most adults choose percutaneous cannulation, while pediatric patients are usually cannulated under general anesthesia by a surgeon.

Firstly, our ECMO experts conducted an initial online assessment of the indications for ECMO in the patients. The ECMO team immediately carried ECMO equipment to the local hospital. After the ECMO team arrived at the local hospital, the patient's condition was immediately re-evaluated, and ECMO installation surgery was performed once the indications for ECMO were confirmed.

After arriving at the local hospital, the patient was re-evaluated and a suitable intubation mode was selected. Among the 22 patients transported to our hospital, 21 was chosen for V-A ECMO mode, and 1 patient experienced hemodynamic instability during treatment and switched from venous-venous mode to V-A mode. The selection of ECMO mode has always been a difficult issue. Some researchers believe that, V-A mode is recommended (11, 15). Fouilloux (5) found that patients with refractory respiratory failure should preferably use V-A ECMO intubation to prevent secondary circulation failure that may occur during transportation.

During the transportation process, it is necessary to carefully monitor children's breathing, heart rate, blood oxygen saturation, pay attention to changes in temperature, blood pressure, and body temperature, and check if the child's position of tracheal intubation has changed or not. After ECMO operation is performed, low temperature blood flows through the extracorporeal tubing. Due to low body temperature can cause chills in children, so it is important to keep warm. And it is recommended to control the temperature of the ECMO water tank between 36.5℃ and 37.0℃, which can be used by temperature control blanket to control the external temperature and prevent hypothermia for children (16). And 5 patients developed multi-organ failure, the reason may be ischemia-reperfusion injury and cytokine storm during ECMO supporting.

Mobile ECMO for inter-hospital transport of pediatric Patients faces risks (3): fluctuations in vital signs: the patient's condition is critical, vital signs are unstable, and the heart, lungs, and blood circulation functions are damaged. Any negligence during the transportation process can be fatal; Risk of complications: The transportation risk is extremely high, and any unexpected event along the way may lead to failure, worsening of the patient's condition, or even death; Monitoring requirements: Patients' vital signs should be continuously monitored, and potential complications should be promptly addressed. We can take the following measures to solve the difficulties: (1) Standardized management can significantly improve transportation efficiency, shorten key times such as personnel arrival, material preparation, task response, and ECMO operations. (2) Rapid response team: it can further shorten patient transportation time, improve transportation efficiency, and effectively reduce the occurrence of adverse events during transportation.

ECMO transport requires the cooperation of members from multiple departments. Establishing an ECMO team in a class-A tertiary hospital to serve the surrounding hospitals can save many pediatric patients with acute and critical illnesses. In addition, improving the technical capacity of the transport is imperative, and we believe that simulation-based training can improve the team's efficiency (13).

## 5 Conclusion

With comprehensive instrumentation support and professional technical team cooperation, it was feasible and safe to apply inter-hospital ECMO transport for critically ill patients, saving precious time and thus, patients' lives.

## Data Availability

The original contributions presented in the study are included in the article/Supplementary Material, further inquiries can be directed to the corresponding authors.

## References

[1] Jenks CL Raman L Dalton HJ. Pediatric extracorporeal membrane oxygenation. Crit Care Clin. (2017) 33(4):825–41. 10.1016/j.ccc.2017.06.005 28887930

[2] Zeng Y Cai Z Xianyu Y Yang BX Song T Yan Q. Prognosis when using extracorporeal membrane oxygenation (ECMO) for critically ill COVID-19 patients in China: a retrospective case series. Crit Care. (2020) 24(1):148. 10.1186/s13054-020-2840-8 32293518 PMC7156900

[3] Kendirli T Kahveci F Özcan S Botan E Sarıcaoğlu C Hasde Aİ Interhospital aircraft/ground extracorporeal membrane oxygenation transportation by a mobile extracorporeal membrane oxygenation team: first Turkish pediatric case series. Turk Arch Pediatr. (2022) 57(6):656–60. 10.5152/TurkArchPediatr.2022.22068 36314958 PMC9682708

[4] Wilhelm MJ Inderbitzin DT Reser D Halbe M Van Tillburg K Albrecht R Outcome of inter-hospital transfer of patients on extracorporeal membrane oxygenation in Switzerland. Swiss Med Wkly. (2019) 149(1516):w20054. 10.4414/smw.2019.20054 30995683

[5] Fouilloux V Gran C Ghez O Chenu C El Louali F Kreitmann B Mobile extracorporeal membrane oxygenation for children: single-center 10 years’ experience. Perfusion. (2019) 34(5):384–91. 10.1177/0267659118824006 30638136

[6] Broman LM Holzgraefe B Palmér K Frenckner B. The Stockholm experience: interhospital transports on extracorporeal membrane oxygenation. Crit Care. (2015) 19:278. 10.1186/s13054-015-0994-6 26160033 PMC4498561

[7] Biscotti M Agerstrand C Abrams D Ginsburg M Sonett J Mongero L One hundred transports on extracorporeal support to an extracorporeal membrane oxygenation center. Ann Thorac Surg. (2015) 100(1):34–9; discussion 9–40. 10.1016/j.athoracsur.2015.02.037 25912741

[8] Bryner B Cooley E Copenhaver W Brierley K Teman N Landis D Two decades’ experience with interfacility transport on extracorporeal membrane oxygenation. Ann Thorac Surg. (2014) 98(4):1363–70. 10.1016/j.athoracsur.2014.06.025 25149055

[9] Dellinger RP Levy MM Rhodes A Annane D Gerlach H Opal SM Surviving sepsis campaign: international guidelines for management of severe sepsis and septic shock: 2012. Crit Care Med. (2013) 41(2):580–637. 10.1097/CCM.0b013e31827e83af 23353941

[10] Leone M Asfar P Radermacher P Vincent JL Martin C. Optimizing mean arterial pressure in septic shock: a critical reappraisal of the literature. Crit Care. (2015) 19(1):101. 10.1186/s13054-015-0794-z 25888071 PMC4355573

[11] Xu Q Jiang X Wang T Zhou Q Wang J Zhang P Study on the extracorporeal membrane oxygenation inter-hospital transport during coronavirus disease 2019 epidemic: based on the transport experience of 6 cases of severe H1N1 influenza virus pneumonia on extracorporeal membrane oxygenation. Zhonghua Wei Zhong Bing Ji Jiu Yi Xue. (2020) 32(4):430–4; Chinese. 10.3760/cma.j.cn121430-20200309-00413 32527347

[12] Nwozuzu A Fontes ML Schonberger RB. Mobile extracorporeal membrane oxygenation teams: the north American versus the European experience. J Cardiothorac Vasc Anesth. (2016) 30(6):1441–8. 10.1053/j.jvca.2016.06.005 27686513 PMC5130610

[13] Allan CK Thiagarajan RR Beke D Imprescia A Kappus LJ Garden A Simulation-based training delivered directly to the pediatric cardiac intensive care unit engenders preparedness, comfort, and decreased anxiety among multidisciplinary resuscitation teams. J Thorac Cardiovasc Surg. (2010) 140(3):646–52. 10.1016/j.jtcvs.2010.04.027 20570292

[14] Broman LM Frenckner B. Transportation of critically ill patients on extracorporeal membrane oxygenation. Front Pediatr. (2016) 4:63. 10.3389/fped.2016.00063 27379221 PMC4904149

[15] Han L Zhang Y Zhang Y Wu W He P. Risk factors for refractory septic shock treated with VA ECMO. Ann Transl Med. (2019) 7(18):476. 10.21037/atm.2019.08.07 31700912 PMC6803222

[16] Moreau A Levy B Annoni F Lorusso R Su F Belliato M The use of induced hypothermia in extracorporeal membrane oxygenation: a narrative review. Resusc Plus. (2023) 13:100360. 10.1016/j.resplu.2023.100360 36793940 PMC9922920

